# Effect of Reperfusion Therapies on Incidence of Early Post-Stroke Seizures

**DOI:** 10.3389/fneur.2021.758181

**Published:** 2021-11-22

**Authors:** Tasnim Mushannen, Rozaleen Aleyadeh, Maria Siddiqui, Maher Saqqur, Naveed Akhtar, Boulenouar Mesraoua, Salman Al Jerdi, Gayane Melikyan, Yanal Shaheen, Haneen Qadourah, Odette Chagoury, Ziyad R. Mahfoud, Naim Haddad

**Affiliations:** ^1^Department of Neurology, Weill Cornell Medicine-Qatar, Ar-Rayyan, Qatar; ^2^Department of Neurology Hamad Medical Corporation, Weill Cornell Medicine-Qatar, Doha, Qatar; ^3^Department of Medicine, Weill Cornell Medicine-Qatar, Ar-Rayyan, Qatar; ^4^Qatar Metabolic Institute, Hamad Medical Corporation, Doha, Qatar; ^5^Department of Population Health Sciences, Weill Cornell Medicine-Qatar, Ar-Rayyan, Qatar

**Keywords:** reperfusion therapies, stroke, early post-stroke seizures, thrombolysis, mechanical thrombectomy, symptomatic seizures

## Abstract

**Objective:** This study aimed to determine the effect of reperfusion therapies on the occurrence of early post-stroke seizures (PSS) in patients with acute ischemic stroke (AIS).

**Background:** Reperfusion therapies are paramount to the treatment of stroke in the acute phase. However, their effect on the incidence of early seizures after an AIS remains unclear.

**Design and Methods:** The stroke database at Hamad Medical Corporation was used to identify all patients who received reperfusion therapies for AIS from 2016 to 2019. They were matched with patients of similar diagnosis, gender, age, and stroke severity as measured by National Institutes of Health Stroke Scale (NIHSS) who did not receive such treatment. The rates of early PSS were calculated for each group.

**Results:** The results showed that 508 patients received reperfusion therapies (342 had IV thrombolysis only, 70 had thrombectomies only, and 96 had received both), compared with 501 matched patients receiving standard stroke unit care. Patients who received reperfusion therapies were similar to their matched controls for mean admission NIHSS score (9.87 vs. 9.79; *p* = 0.831), mean age (53.3 vs. 53.2 years; *p* = 0.849), and gender distribution (85 vs. 86% men; *p* = 0.655). The group receiving reperfusion therapies was found to have increased stroke cortical involvement (62 vs. 49.3%, *p* < 0.001) and hemorrhagic transformation rates (33.5 vs. 18.6%, *p* < 0.001) compared with the control group. The rate of early PSS was significantly lower in patients who received reperfusion therapies compared with those who did not (3.1 vs. 5.8%, respectively; *p* = 0.042). When we excluded seizures occurring at stroke onset prior to any potential treatment implementation, the difference in early PSS rates between the two groups was no longer significant (2.6 vs. 3.9%, respectively; *p* = 0.251). There was no significant difference in early PSS rate based on the type of reperfusion therapy either (3.2% with thrombolysis, 2.9% with thrombectomy, and 3.1% for the combined treatment, *p* = 0.309).

**Conclusions:** Treatment of AIS with either thrombectomy, thrombolysis, or both does not increase the risk of early PSS.

## Introduction

Annually, 15 million people develop a new stroke worldwide, with many developing post-stroke complications (1). One of these complications is early post-stroke seizures (PSS). Early PSS is defined as seizure(s) occurring within 7 days of stroke. Seizures occurring more than 1 week after stroke are considered late PSS or post-stroke epilepsy. Early PSS occurs in 2.2–6.5% of patients with ischemic stroke (2, 3). Early PSS is associated with an increased risk of developing epilepsy, greater disability, longer hospital stay, and increased mortality risk (4–7).

The first-line treatment modalities for acute ischemic stroke include reperfusion therapies, namely thrombolysis with tissue plasminogen activator (tPA) and/or mechanical thrombectomy. The use of tPA or thrombectomy is limited by time constraints, and both have multiple contraindications (8, 9). Patients who do not qualify for reperfusion therapies receive standard stroke unit care (antiplatelet agents, cholesterol-lowering medications, treatment of underlying stroke risk factors, rehabilitation, etc.) (9).

Several trials, including Multicenter Randomized Clinical Trial of Endovascular Treatment for Acute Ischemic Stroke in the Netherlands (MR CLEAN), The Endovascular Treatment for Small Core and Anterior Circulation Proximal Occlusion with Emphasis on Minimizing CT to Recanalization Times (ESCAPE), and Revascularization with Solitaire FR Device versus Best Medical Therapy in the Treatment of Acute Stroke Due to Anterior Circulation Large Vessel Occlusion Presenting within Eight Hours of Symptom Onset (REVASCAT), have proven the benefit of thrombectomy on residual neurological deficits (10–12). Similar trials were conducted for thrombolysis, most notably Alteplase Thrombolysis for Acute Noninterventional Therapy in Ischemic Stroke (ATLANTIS), The European Cooperative Acute Stroke Study (ECASS), and National Institute of Neurological Disorders and Stroke rt-PA Stroke Study Group (NINDS) trials (13–15). The effect of reperfusion therapies on early PSS, in particular, is a seldomly studied question. Most studies have small sample sizes, and the results are mixed showing decreased (16), increased (17, 18), or no effect (5, 19) of initial treatment modality on the incidence of early PSS. Some studies theorize that reperfusion therapies increase the risk of seizures, as treated patients tend to present with more hemorrhagic transformation, a strong risk factor for the development of early PSS (20, 21). Other pre-clinical studies have shown that overexpression of endogenous tPA lowers the seizure threshold in mice (22). However, recent studies by Lekoubou et al. and Zollner et al. showed no increased risk of early PSS following reperfusion therapy (5, 23). The aim of our study was to further elucidate the association between reperfusion therapies (tPA, thrombectomy, or both) and the incidence of early PSS.

## Methods

This study was approved by the Institutional Review Board at Hamad Medical Corporation (HMC) and Weill Cornell Medicine–Qatar. Individual written consent was waived as the data was collected from a stroke database and through a retrospective chart review.

In this matched case-control study, subjects who received tPA and/or thrombectomy for an ischemic stroke between 2016 and 2019 were identified from the HMC Stroke Database. The control group consists of patients with a similar diagnosis who did not qualify for reperfusion therapies and just received standard stroke unit care, matched for age, gender, and stroke severity [National Institutes of Health Stroke Scale (NIHSS) score] using propensity score. HMC is the largest health system in Qatar and cares for almost all stroke patients in the country; thus, the HMC Stroke Database is equivalent to a national registry. Subjects were subsequently excluded if they had a pre-existing diagnosis of epilepsy or if the final diagnosis was later determined to be a stroke mimic or transient ischemic attack.

The following variables were collected from the database and reverified by scrutinizing the medical files of the actual patients: age, gender, history of a prior stroke, NIHSS score on admission, treatment of the ischemic stroke (tPA only, thrombectomy only, tPA and thrombectomy, or standard stroke unit care), modified Rankin Scale (mRS) score at discharge, TOAST classification for stroke etiology, presence of cortical involvement in the infarcted area, presence of radiological hemorrhagic transformation, development of seizure(s) within 7 days of stroke onset, and latency/time to first early seizure. All seizures reported in the medical records were counted, regardless of type or classification.

All statistical analyses were conducted using IBM SPSS version 26 (IBM Corp. Armonk, New York, NY, USA), and *p* < 0.05 was considered statistically significant. For descriptive statistics, *M* and *SD* were used for quantitative data, and percentages were used for qualitative data. Univariate analysis of quantitative data was performed with Student's independent *t*-test. Univariate analysis of qualitative data was performed with chi-square and/or Fisher's exact test.

## Results

Among the total 1,009 patients who were retained and had their data analyzed, 508 received reperfusion therapy (342 tPA only, 70 thrombectomies only, and 96 tPA and thrombectomy) and 501 control patients received just standard stroke unit care. Patients who received reperfusion therapies were similar to their matched controls for mean NIHSS score (9.87 vs. 9.79; *p* = 0.831), mean age (53.3 vs. 53.2 years; *p* = 0.849), and gender distribution (85 male and 15% female vs. 86 male and 14% female; *p* = 0.655) (Table 1). The rate of early PSS was lower in patients who received reperfusion therapies compared with those who received standard stroke unit care (3.1 vs. 5.8%; *p* = 0.042). This was noted despite the observation that hemorrhagic transformation and stroke cortical involvement, wherein two established risk factors for early PSS, were significantly greater in the reperfusion therapy group (*p* < 0.001 for both) (Table 1).

**Table 1:** Characteristics of patients with ischemic stroke receiving reperfusion treatment or standard stroke unit care.

		**Reperfusion therapy (*****n*** **=** **508)**		**Standard stroke unit care (*****n*** **=** **501)**		***P*-value**
		**Count/mean**	**Percentage/SD**	**Count/mean**	**Percentage/SD**	
Age (Mean, SD)		53.3	13.1	53.2	14	0.849
Sex	Male	432	85.0%	431	86.0%	0.655
	Female	76	15.0%	70	14.0%	
NIHSS (mean, SD)		9.87	6	9.79	6.46	0.831
Early PSS		16	3.1%	29	5.8%	**0.042**
Cortical involvement		315	62.0%	247	49.3%	**<0.001**
Hemorrhagic transformation		170	33.5%	93	18.6%	**<0.001**

*The bold values represent p-values that reached statistical significance*.

**Figure 1:**
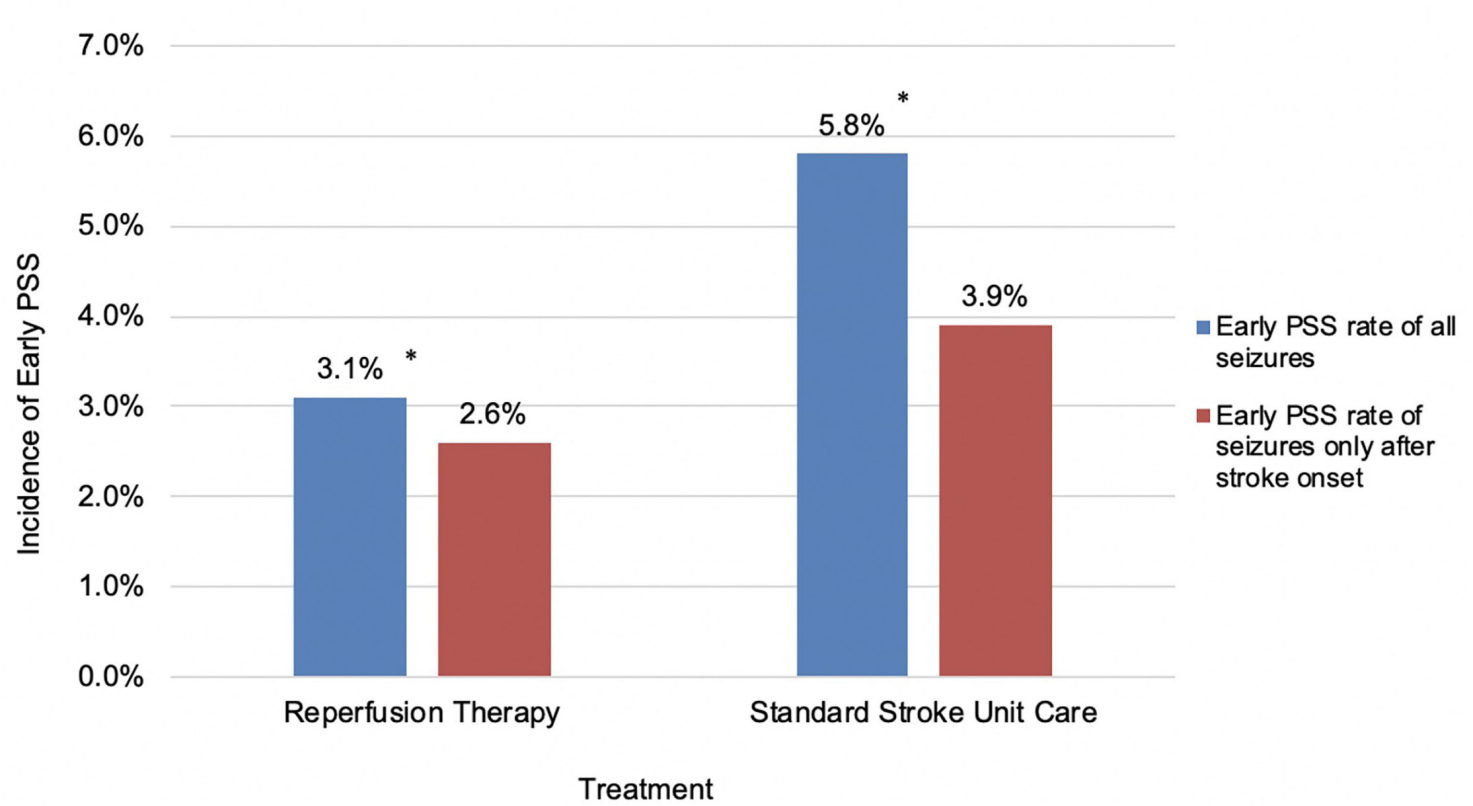
Incidence of early PSS in patients receiving reperfusion therapy or standard stroke unit care. *P < 0.05.

**Figure 2:**
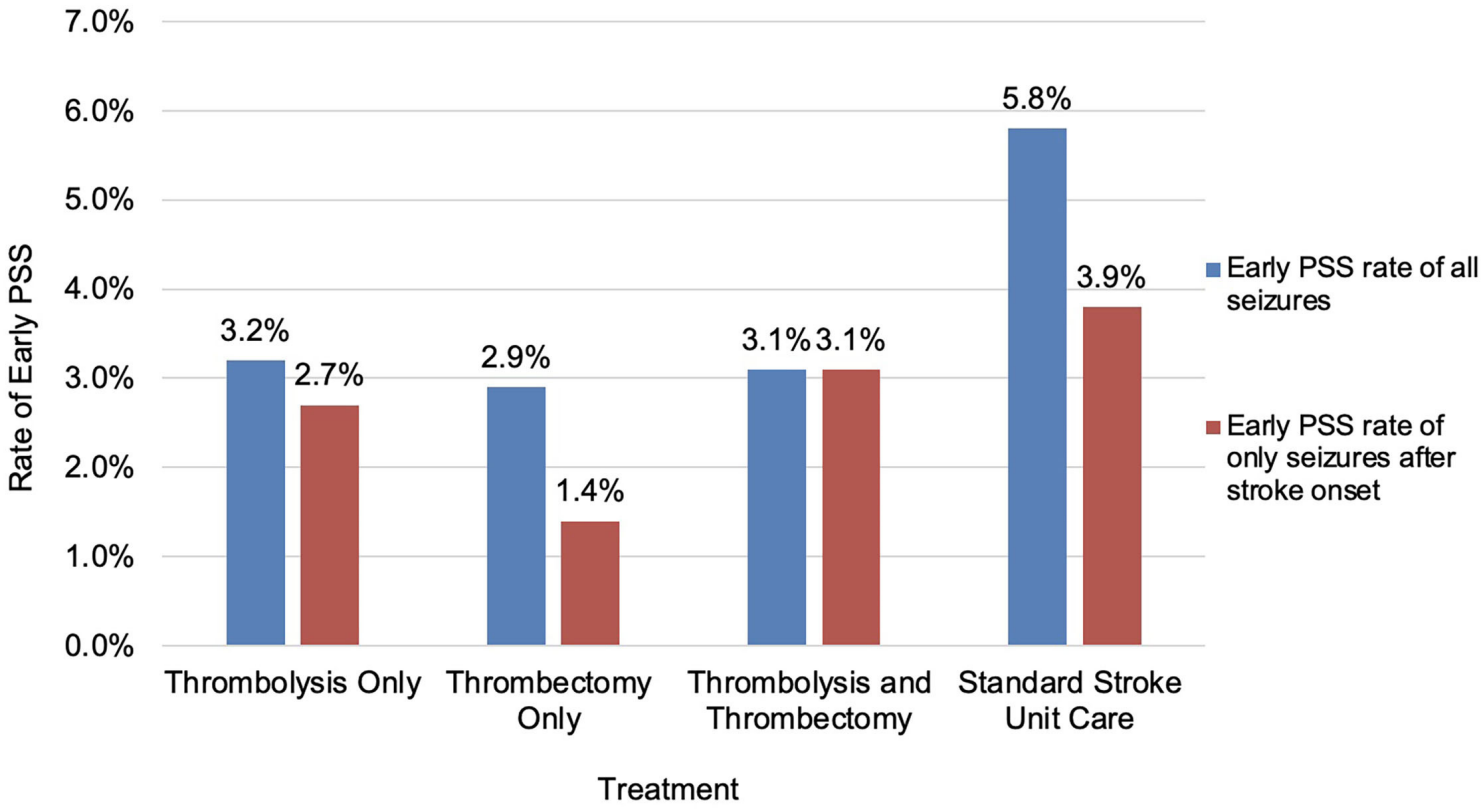
Rates of early PSS based on treatment type.

There was no significant difference in the incidence of early PSS based on the type of reperfusion therapy (Table 2). As we were analyzing the latency between stroke onset and seizures, it became obvious that some seizures (28%) occurred at stroke onset and clearly before treatment decisions or implementation. For better precision in isolating the effect of treatment on early seizures, we recalculated an early PSS rate excluding events noted at stroke onset and prior to the arrival to the hospital. For seizures that occurred exclusively after stroke onset, the difference in early PSS rates between the two groups was no longer significant (2.6% in the reperfusion therapy group vs. 3.9% in the control group; *p* = 0.251) (Figures 1, 2).

**Table 2:** Rates of early PSS, hemorrhagic transformation, and stroke cortical involvement in each treatment group.

	**tPA (*****n*** **=** **342)**		**Thrombectomy (*****n*** **=** **70)**		**Both (*****n*** **=** **96)**		***P*-value**
	**Count**	**Percentage**	**Count**	**Percentage**	**Count**	**Percentage**	
Early PSS	11	3.20%	2	2.90%	3	3.10%	0.999
Hemorrhagic transformation	94	27.50%^a^	34	48.60%^b^	42	43.80%^b^	**<0.001**
Stroke cortical involvement	180	52.6%^a^	60	85.7%^b^	75	78.10%^b^	**<0.001**

ab *Groups with similar letters are not statistically different while those with different letters are. The bold values represent p-values that reached statistical significance*.

A secondary analysis looked at variables compared between patients who developed early PSS and patients who did not, regardless of treatment. This included age, NIHSS on admission, mRS upon discharge, and TOAST+ criteria (Table 3). On average, patients who developed early PSS were older than those who did not (57.8 vs. 53 respectively; *p* = 0.021). The stroke severity was higher in patients with seizures (mean NIHSS score in patients with early PSS: 13.4 vs. with no early PSS: 9.7, *p* < 0.001). As for the effect of stroke mechanism, the rate of early PSS was highest in patients with stroke of undetermined etiology, followed by cardioembolic stroke, large vessel stroke, small vessel disease, and stroke due to other determined etiology (10, 5.4, 5, 2.8, and 1.2% respectively; *p* = 0.034). The statistical difference stems from the relatively higher rate of PSS in the stroke of unknown etiology vs. lower rates in the stroke of other determined etiology and small vessel disease. A higher mRS, indicating a poorer functional outcome, was noted on discharge after the development of early PSS when compared with patients who did not develop early PSS (4.32 vs. 2.84 respectively; *p* < 0.001). However, the difference in mRS score of 6 and thus mortality between the seizure (9.1%) and non-seizure (4.3%) groups did not reach statistical significance (*p* = 0.134). In patients who developed early PSS, 55.8% of seizures occurred within the first day and 83.7% of seizures occurred within the first 72 h.

**Table 3:** Age, NIHSS, TOAST criteria of ischemic stroke, and mRS score upon discharge comparison between seizure and non-seizure groups.

		**Early PSS (*****n*** **=** **45)**		**No early PSS (*****n*** **=** **964)**		***P*-value**
		**Count/mean**	**Percentage/SD**	**Count/mean**	**Percentage/SD**	
Age		57.8	15.8	53.0	13.4	**0.021**
NIHSS		13.4	7.2	9.7	6.1	**<0.001**
mRS		4.32	1.34	2.84	1.70	**<0.001**
mRS = 6		4	9.1%	41	4.3%	0.134
TOAST+	EMB	10	5.4%	174	94.6%	**0.034** ^†^
	LVD	17	5.0%	320	95.0%	
	SDE	1	1.2%	84	98.8%	
	SUE	8	10.0%	72	90.0%	
	SVD	9	2.8%	314	97.2%	

*EMB, cardioembolic etiology of stroke; LVD, large vessel disease etiology of stroke; SDE, stroke due to other determined etiology; SUE, stroke of unknown origin; SVD, small vessel disease etiology of stroke*.

† *Differences in early PSS rates were significant between the SUE group and each of the SVD and SDE groups. The bold values represent p-values that reached statistical significance*.

## Discussion

Our study is an attempt to clarify the controversy surrounding the effect of reperfusion therapies on rates of early PSS. We found no evidence of increased risk of early seizures following reperfusion therapies for ischemic stroke. The possibility of a causal effect between these treatments and the occurrence of seizures stems initially from some pre-clinical hypotheses. tPA was postulated to act as a proconvulsant since it upregulates the excitatory N-methyl-D-aspartate (NMDA) receptors in the brain, thereby increasing the ability to induce a hyperexcited neuronal state (22, 24). Studies also show that mice with overexpression of endogenous tPA have lower seizure thresholds (24). Reperfusion injury through oxygen-free radicals and/or calcium overload is another hypothesis for how tPA/thrombectomy may increase seizure risk (24, 25). Another frequently discussed explanation for the association between reperfusion therapy and seizures is that tPA/thrombectomy increases the risk of hemorrhagic transformation, an established risk factor for seizures (24, 25). Nevertheless, our study shows that although reperfusion therapies increase the risk of hemorrhagic transformation, they have no measurable effect on the risk of early PSS.

Other studies argue that tPA/thrombectomy is protective against seizures by reducing the size of infarction, which is also an established risk factor for seizures (24). These reperfusion therapies also reduce the clinical and imaging severity of ischemic strokes, two additional established risk factors for post-stroke seizures (24). Thus, the existing literature presents evidence for and against increased seizure risk from reperfusion therapy.

Our finding that reperfusion therapy does not increase the risk of early PSS agreed with some clinical studies and disagreed with others. A large, recent study by Zollner et al. found no increased risk of acute seizures in patients treated with intravenous thrombolysis (13,356 patients) or intravenous thrombolysis with mechanical thrombectomy (1,013 patients) after matching for age, stroke severity, and pre-stroke function (23). Another large systematic review and meta-analysis by Lekoubou et al. determined that the rate of early PSS was similar between patients who received tPA and patients who did not (5). A prospective, multicenter study by Belcastro et al. found no significant difference in the rate of early PSS in 262 patients who received tPA compared with 254 patients who did not (19). Another prospective study also found no significant difference in the rate of early PSS in 101 patients treated with tPA and 50 patients treated without tPA (26).

However, in the study by Alvarez et al., thrombolysis with tPA was associated with an increased risk of early PSS. In this study, 28 patients developed early seizures, 11 of whom received tPA. The control group consisted of 100 randomly selected patients with ischemic strokes who did not develop early seizures, and no matching was done. NIHSS score was not controlled for during this analysis (17). Because the selection of patients for tPA treatment is also influenced by stroke severity, which is related to NIHSS score, NIHSS score may have been a confounding variable that was not accounted for when determining the association between tPA and early PSS. In a case-control study by Brigo et al. with 79 cases (patients who developed seizures) and 158 controls (patients who did not develop seizures), thrombolysis with tPA was an independent risk factor for early PSS. However, the NIHSS score was not controlled for in this analysis either (27).

As for mechanical thrombectomy, most studies estimate early PSS rates without comparison to a control group with the exception of the previously mentioned study by Zollner et al. The latter did include mechanical thrombectomy and a control group, and they found no increased risk of early PSS (23). The study of Brigo et al. recently published a large retrospective study that included reperfusion therapies other than IV tPA. The rate of early PSS following all reperfusion therapies was 4.71%, and the rate for specifically intra-arterial thrombolysis with or without mechanical thrombectomy was 5.74%. Although there was no control group to statistically test the association between reperfusion therapies and early PSS, they noted that the 4.71% rate is within range of the overall incidence of early PSS after ischemic stroke regardless of treatment type (28). Other studies reported 2.4, 4.4, and 6.1% rates of early seizures among the respective 459, 90, and 344 patients who received thrombectomy, respectively (25, 29, 30). Agashe et al. investigated the rate of early PSS after thrombectomy beyond 6 h in the extended time window specifically and found it to be grossly similar at 4%. A study by Naylor et al. determined that reperfusion therapies, including thrombectomy, were associated with increased risk of post-stroke epilepsy, but they did not study the association with early seizures (18).

Many studies suggest a less favorable functional outcome in patients receiving either reperfusion treatments if the course is complicated by early PSS (17, 25, 31–36). It is indeed relieving to know that there is no indirect decline in the benefit from these treatments through an undesirable increase in seizures.

Our study matched patients for NIHSS score and age to control for some known PSS risk factors and better isolate the effect of stroke treatment. The case-control design of the study was chosen because early PSS is a relatively uncommon complication of ischemic stroke. We specifically separated in our analysis the seizures occurring at stroke onset as they cannot be attributed to the treatment modality. We did not suffice ourselves with the registry data on seizure occurrence, but we scrutinized the actual medical reports for such mention. Not all seizures were accounted for in the database, thus we avoided outcome underreporting. However, our project has limitations. The use of EEG was rather scarce. This may lead to underestimating the rate of early PSS, as some seizures present with confusion, behavior changes, or subtle sensory changes, which often go undetected in patients already experiencing major deficits (6). Further characterization of the seizures (i.e., detailed semiology, classification, etc.) was not consistently available and thus could not be analyzed. A prospective study with video-EEG assessment could help overcome these limitations.

In conclusion, this study has provided further evidence that reperfusion therapies do not increase the risk of early PSS. Reperfusion therapies are the mainstay of treatment for ischemic stroke, and this study supports that the concern of pro-convulsive effects of reperfusion therapies should not be prioritized over the substantial benefit that reperfusion therapies provide. This study has also shown that the specific type of reperfusion therapy does not affect the rate of early seizures, but further studies with larger sample sizes under each type of reperfusion therapy are needed to assess the association between each reperfusion therapy and early PSS.

## Data Availability Statement

The original contributions presented in the study are included in the article/supplementary material, further inquiries can be directed to the corresponding author/s.

## Ethics Statement

The studies involving human participants were reviewed and approved by Institutional Review Board at Hamad Medical Corporation and Weill Cornell Medicine–Qatar. Written informed consent for participation was not required for this study in accordance with the national legislation and the institutional requirements.

## Author Contributions

All authors listed have made a substantial, direct, and intellectual contribution to the work and approved it for publication.

## Conflict of Interest

The authors declare that the research was conducted in the absence of any commercial or financial relationships that could be construed as a potential conflict of interest.

## Publisher's Note

All claims expressed in this article are solely those of the authors and do not necessarily represent those of their affiliated organizations, or those of the publisher, the editors and the reviewers. Any product that may be evaluated in this article, or claim that may be made by its manufacturer, is not guaranteed or endorsed by the publisher.
